# The effect of increases in physical activity on oral health and function in older adults in Singapore: A longitudinal causal inference study

**DOI:** 10.1016/j.jnha.2026.100968

**Published:** 2026-08-28

**Authors:** John Rong Hao Tay, Gustavo G. Nascimento, Rahul Malhotra, Pedro C. Hallal, Marco A. Peres

**Affiliations:** a Health Services Research & Population Health Programme, Duke-NUS Medical School, Singapore, 8 College Rd, Singapore 169857, Singapore; b Department of Restorative Dentistry, National Dental Centre Singapore, 5 Second Hospital Ave, Singapore 168938, Singapore; c National Dental Research Institute Singapore, National Dental Centre Singapore, 5 Second Hospital Ave, Singapore 168938, Singapore; d School of Dentistry, University of Utah, 530 S Wakara Way, Salt Lake City, UT 84108, United States; e Centre for Ageing Research & Education, Duke-NUS Medical School, Singapore, 8 College Rd, Singapore 169857, Singapore; f Department of Health and Kinesiology, University of Illinois Urbana-Champaign, 1206 S. Fourth Street, Champaign, IL 61820, United States; g Oral Health Academic Clinical Programme, Duke-NUS Medical School, Singapore, 5 Second Hospital Ave, Singapore 168938, Singapore

**Keywords:** Aging, Epidemiology, Oral health, Mastication, Exercise, Causal inference, Public health

## Abstract

**Background:**

Physical activity may benefit oral health, but causal evidence is lacking. This study estimated the effect of modifying physical activity on self-rated oral health and masticatory function impairment in older adults using longitudinal modified treatment policies.

**Methods:**

Data from three waves (2016–2024) of THE SIGNS Study, a nationally representative cohort of community-dwelling adults aged 60 years and above in Singapore (*N* = 3981), were analysed. Ten physical activity scenarios were emulated, including upward, downward, and stochastic shifts in weekly metabolic equivalent (MET) minutes (WHO guideline: ≥600MET-min/week). Causal parameters were estimated using targeted maximum likelihood estimation with Super Learner.

**Results:**

For poor self-rated oral health, raising all participants to at least 450 MET-min/week (equivalent to 112.5 min per week of moderate-intensity physical activity) produced the largest risk reduction (RR: 0.88, 95% CI: 0.82–0.95). Even raising participants below 150 MET-min/week toward 150 MET-min/week reduced risk by 7% (RR: 0.93, 95% CI: 0.88-0.99). For masticatory function impairment, reducing physical activity increased risk in a dose-response manner: eliminating all activity increased risk by 21% (RR: 1.21, 95% CI: 1.10-1.33), while raising 50% of inactive participants toward 600 MET-min/week reduced risk by 6% (RR: 0.94, 95% CI: 0.90–0.99). A stricter masticatory function impairment definition produced consistent but stronger effects. Estimates were robust across estimators and under extreme attrition assumptions. Increasing physical activity may reduce the risk of poor self-rated oral health, while reducing physical activity may increase masticatory function impairment.

**Conclusions:**

These findings support integrating physical activity promotion within oral health strategies for ageing populations.

## Highlights

• Increasing physical activity lowered poor self-rated oral health in older adults.
• Self-rated oral health gains plateaued beyond 450 MET-min/week of activity.
• Reducing activity worsened masticatory function in a dose-response manner.
• Eliminating all activity raised masticatory impairment risk by 21%.

## 1 Introduction

Physical activity is a central determinant of healthy ageing with well-documented effects on metabolic regulation, cognitive function, and physiological resilience [[1], [2], [3], [4]]. Older adults who engage in higher levels of physical activity are more likely to remain free of major chronic diseases in later life [1]. Regular physical activity also preserves metabolic health and neuromuscular efficiency, reducing the likelihood of frailty-related functional loss [5,6]. On the other hand, physical inactivity has been associated with diminished appetite and poorer nutritional status, both of which contribute to frailty development [7].

These systemic consequences of physical inactivity have implications for oral health. Physical activity reduces circulating levels of pro-inflammatory cytokines, including C-reactive protein, interleukin-6, and tumour necrosis factor-α, which are implicated in periodontal tissue destruction [8]. Regular physical activity additionally improves glycaemic control and reduces adiposity, both established risk factors for periodontal disease [9,10]. Physical activity may also influence the oral environment directly: regular moderate exercise has been associated with higher salivary flow rates and elevated salivary antimicrobial proteins, both of which support oral mucosal defence [11]. Physical inactivity may impair neuromuscular coordination and reduce masticatory muscle strength, thereby limiting masticatory efficiency [12,13]. Behavioural pathways may reinforce these effects: physically active individuals cluster with other health-promoting behaviours, including healthier dietary patterns and non-smoking behaviours [14] whereas mobility limitations linked to physical inactivity are associated with reduced utilisation of dental services and greater likelihood of untreated oral conditions [15].

Physical activity thus represents an upstream, modifiable behaviour that may influence oral health through inflammatory, neuromuscular, and behavioural pathways. This study hypothesises that increasing physical activity in older adults reduces the risk of poor self-rated oral health and masticatory function impairment. As even modest increments in physical activity can confer measurable health benefits [16], we defined population-level interventions that increase weekly physical activity within feasible bounds for older adults. The aim of this study is to estimate the causal impact of modifying physical activity on self-rated oral health and masticatory function impairment, under a series of upward, downward, and stochastic shifts in activity.

## 2 Methods

### 2.1 Study sample

This study used data from the Transitions in Health, Employment, Social Engagement and Inter-generational Transfers in Singapore Study (THE SIGNS Study; hereafter, TSS). TSS is a nationally representative longitudinal survey of 4549 older Singapore citizens and permanent residents, aged 60 years and above, at baseline, first conducted in 2016–2017 [17]. Three waves of TSS data collected in 2016–2017, 2019–2020, and 2023–2024 were analysed, providing up to nine years of follow-up for participants. The IRB at the National University of Singapore approved the study protocol (Reference Code: B-15-152; NUS-IRB-2022-627).

### 2.2 Measures

Physical activity was assessed at Wave 1 (baseline, 2016–2017) and Wave 2 (2019–2020), both preceding ascertainment of the primary outcomes at Wave 3 (2023–2024). The exposure was total weekly metabolic equivalent (MET) minutes of physical activity, calculated from self-reported frequency and duration of physical activity in a typical week across work (paid and unpaid, including household chores or activities), transport (walking and/or cycling for commuting to and from places), and recreation (sports, fitness, or leisure activities using the WHO Global Physical Activity Questionnaire (GPAQ). Standard quality checks were applied [18] and participants reporting values above the 95th percentile (>6000 MET-min/week) were excluded (Appendix S1). To ensure feasible intervention targets, participants were stratified by age band (60–79, ≥80) and Activity of Daily Living (ADL) limitations (none vs ≥1). Within each stratum, the 90th percentile of MET-min/week was estimated, which served as an upper limit for intervention-assigned activity levels (Appendix S1).

Oral health was operationalised using two complementary self-reported dimensions: (i) self-rated oral health, and (ii) masticatory function. Self-rated oral health is an indicator of overall oral status and predictive of clinically assessed oral conditions such as dental pain, tooth mobility, and xerostomia [19,20], while masticatory function is linked to the number and distribution of functional teeth, oral diadochokinesis (dexterity of tongue and lip motor function), and diet quality in older adults [[21], [22], [23]].

The primary outcomes, assessed at Wave 3, were: (1) poor self-rated oral health, derived from the item “Over the past 6 months, how would you rate your state of oral health?” with response options excellent, very good, good, fair, and poor. Responses were dichotomised as poor (fair/poor) versus good (excellent/very good/good) [24]; (2) Any masticatory function impairment, derived from a single item asking participants to identify the hardest food group they could bite and chew, from a six-level scale ranging from the toughest foods (e.g., dried anchovies) to the softest (e.g., bananas, hard-boiled egg), or inability to chew any listed food [25]. Responses were dichotomised as no impairment (able to chew the hardest group) versus any impairment (all other responses). A stricter definition classified impairment as difficulty with foods of moderate toughness or below (Groups 3-6 versus Groups 1-2) (Appendix Table 1).

**Fig. 1:**
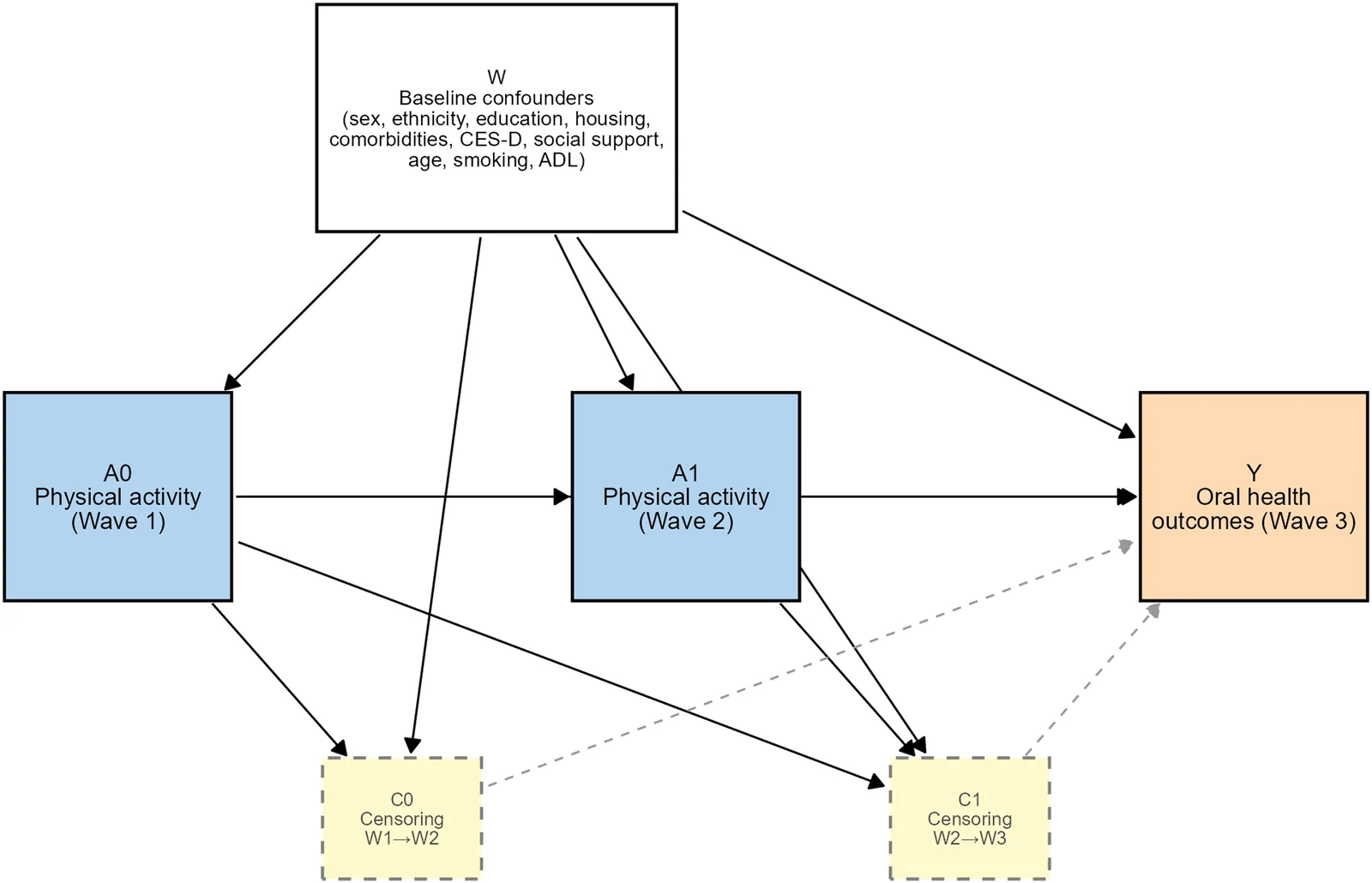
Directed acyclic graph of the hypothesised causal structure. W denotes all time-invariant baseline confounders (sex, ethnicity, education, housing, comorbidities, depressive symptoms, social support, age, smoking status, and ADL limitation). A0 and A1 denote physical activity at Wave 1 and Wave 2 (exposure). Y denotes oral health outcomes at Wave 3. C0 and C1 are censoring indicators between waves; dashed arrows indicate that outcome ascertainment is conditional on remaining uncensored. Wave 2 covariates were not included as time-varying confounders because they may lie on the causal pathway between physical activity and oral health outcomes.

Confounders included sociodemographic, health, and psychosocial characteristics measured at baseline (Appendix S2, Fig. 1).

### 2.3 Statistical analysis

**Fig. 2:**
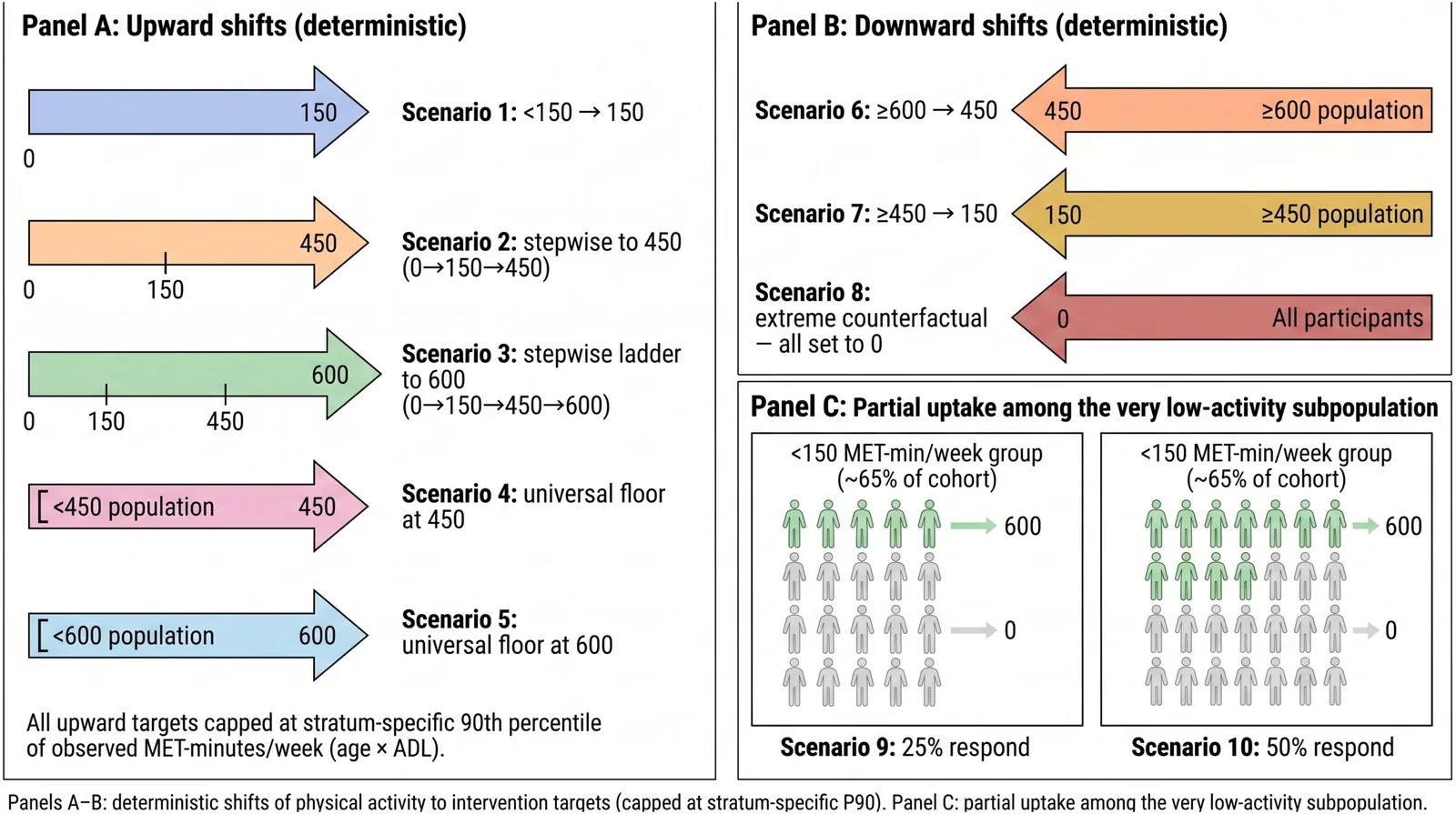
Schematic of the 10 hypothetical physical activity scenarios. Panel A: deterministic upward shifts (Scenarios 1–5). Panel B: deterministic downward shifts (Scenarios 6–8). Panel C: partial uptake among very inactive participants (Scenarios 9–10). All upward targets are capped at stratum-specific 90th percentile of observed MET-min/week (age x ADL).

A series of hypothetical physical activity scenarios were emulated to estimate their effects on poor self-rated oral health and masticatory function impairment. At each wave, an individual’s observed weekly MET-minutes were shifted to new levels defined by ability-adjusted treatment policies using the longitudinal modified treatment policies (LMTPs) framework [26]. The same rule was applied at both waves, representing a sustained shift in the population-level exposure distribution rather than a one-time change at baseline. Counterfactual outcomes under each scenario were compared against the observed exposure. Ten hypothetical intervention scenarios were evaluated, grouped into stepwise minimum-threshold increases (Scenarios 1–3), universal minimum-activity policies (Scenarios 4–5), reductions in physical activity (Scenarios 6–8), and partial-uptake interventions (Scenarios 9–10) (Fig. 2). Physical activity interventions in older adults have achieved median increases of approximately 285–475 MET-min/week at moderate intensity, with the most effective interventions producing larger gains [27]. Scenarios involving higher activity levels represent extreme counterfactuals designed to characterise the dose-response relationship while stochastic scenario model partial uptake consistent with achievable population-level change [2].

Scenario thresholds of 600, 450, and 150 MET-min/week were used, corresponding to 150, 112.5, and 37.5 min moderate-intensity-equivalent minutes per week (vigorous-intensity minutes are weighted double relative to moderate-intensity minutes [18]), and where 600 MET-min/week corresponds to the WHO guidelines minimum of 150 min of moderate-intensity or 75 min of vigorous-intensity activity weekly [16]. Participants were classified accordingly as meeting guidelines (≥600), approaching guidelines (450–599), below guidelines (150–449), or below any guideline threshold (<150).

For all scenarios involving increases in physical activity (Scenarios 1–5, 9–10), intervention targets were capped at each participant's age-band- and ADL-specific 90th percentile of observed MET-min/week. Where the stated scenario target (e.g., 600 MET-min/week) exceeded a participant’s stratum-specific cap, the participant was raised to the cap rather than the target. For reduction scenarios (Scenarios 6-8), no cap was applied.

#### 2.3.1 Stepwise minimum-threshold interventions

1: “What if very inactive participants (<150 MET-min/week) were raised toward 150 MET-min/week?”

2: “What if very inactive participants (<150 MET-min/week) were raised toward 150 MET-min/week and moderately active participants (150–449 MET-min/week) were raised toward 450 MET-min/week?”

3: “What if all participants below guideline levels were raised toward 600 MET-min/week through sequential steps: <150 → 150, 150–449 → 450, and 450–599 → 600 MET-min/week?”

#### 2.3.2 Universal minimum-activity interventions

4: “What if all participants were raised to at least 450 MET-min/week?”

5: “What if all participants were raised to at least 600 MET-min/week?”

#### 2.3.3 Reductions in physical activity

6: “What if high activity (≥600 MET-min/week) were reduced to 450 MET-min/week?”

7: “What if all moderate-to-high activity (≥450 MET-min/week) were reduced to 150 MET-min/week?”

8: “What if all physical activity were set to zero (extreme counterfactual)?”

#### 2.3.4 Partial-uptake interventions

Two partial-uptake scenarios were additionally defined [2], in which only a random subset of very inactive participants (<150 MET-min/week) achieved guideline-level activity while the remainder continued as observed. We defined uptake proportions of 25% and 50% to characterise the dose-response relationship across a range of plausible implementation levels.

Uptake status was assigned via a fixed baseline uniform random draw per participant, so that the same individuals were considered “takers” at both waves:

9: “What if 25% of very inactive participants (<150 MET-min/week) were raised toward 600 MET-min/week?”

10: “What if 50% of very inactive participants (<150 MET-min/week) were raised toward 600 MET-min/week?”

Statistical parameters for each intervention scenario were estimated using targeted maximum likelihood estimation (TMLE), which provides doubly robust estimates by integrating g-computation with inverse probability weighting to adjust for confounding and censoring [28]. Censoring probabilities at each wave were estimated within the TMLE procedure using the same Super Learner ensemble and incorporated as inverse probability of censoring weights. Flexible, nonparametric estimation was achieved using the Super Learner algorithm [29], combining generalized linear models, generalized additive models, and penalized regression (elastic net) through cross-validated model stacking. Missing data were imputed using chained equations (10 datasets) [30] and pooled using Rubin’s rules. Attrition was accounted for via inverse probability of censoring weights. Intervention effects were estimated using absolute risk differences (RDs) and risk ratios (RRs) with 95% confidence intervals, comparing each shifted scenario with the observed exposure. E-values were computed to assess the robustness of these estimates to potential unmeasured confounding [31]. Both Super Learner and generalised linear model (GLM)-only estimators were fitted to assess concordance between the flexible and parametric approaches. All analyses were conducted in R (version 4.4.2), and the *lmtp* package was used.

## 3 Results

Of 4,549 participants, 4 were excluded due to invalid physical activity data and 564 were excluded for reporting physical activity above the 95th percentile (>6000 MET-min/week), yielding an analytic sample of 3981 participants at Wave 1 (2016–17). Of these, 2383 were followed up at Wave 2 (2019–20) and 1219 at Wave 3 (2023–24), with 1598 and 1164 participants lost to follow-up between waves, respectively (Appendix Fig. 1). The mean age at baseline was 73.2 (SD 8.5) years, with 56.2% female and 73.7% Chinese participants (Table 1, Appendix Table 2–4). Most participants (62.3%) had a primary school education or below. Physical activity was bimodally distributed: 64.9% reported less than 150 MET-min/week, while 25.9% met WHO guidelines of at least 600 MET-min/week, with a mean of 517.9 (1004.6) MET-min/week. Clinically significant depressive symptoms were present in 12.7%, diabetes mellitus in 27.8%, and cardiovascular disease in 15.1% of participants.

**Table 1:** Baseline (Wave 1) characteristics of study participants.

Characteristic	Overall (*N* = 3981)
Age, years	73.2 (8.5)
Sex	
Male	1744 (43.8)
Female	2237 (56.2)
Ethnicity	
Chinese	2935 (73.7)
Non-Chinese	1046 (26.3)
Education	
Primary school or below	2481 (62.3)
Above primary school	1500 (37.7)
Housing type	
1–2 room government-built	338 (8.5)
3 room government-built	968 (24.3)
4–5 room government-built/private	2674 (67.2)
Smoking status	
Never or former	3568 (89.6)
Current	413 (10.4)
ADL limitation	
None	3463 (87.0)
≥1 limitation	518 (13.0)
Social network scale (Lubben, 0–60)	25.7 (11.1)
Clinically significant depressive symptoms	505 (12.7)
Diabetes mellitus	1105 (27.8)
Cardiovascular disease	602 (15.1)
Cerebrovascular disease	224 (5.6)
Cancer	192 (4.8)
Physical activity, MET-min/week	
Mean (SD)	517.9 (1004.6)
<150	2585 (64.9)
150–599	363 (9.1)
≥600 (meeting WHO guideline)	1033 (25.9)

Abbreviations: ADL, activity of daily living; CES-D, Center for Epidemiologic Studies Depression Scale; MET, metabolic equivalent of task; N, number; SD, standard deviation; WHO, World Health Organization.

Footnote: Values are *N* (%) for categorical variables and Mean (SD) for continuous variables. Statistics are pooled across 10 imputed datasets. Clinically significant depressive symptoms defined as CES-D 11-item score ≥7 [51,52].

In the context of the risk of poor self-rated oral health, the largest reduction among all scenarios was observed for Scenario 4 – if all participants accumulated at least 450 MET-min/week of physical activity, the risk was 12% lower (RR: 0.88, 95% CI: 0.82–0.95; E-value 1.53). This corresponded to a risk difference of −4.9 percentage points (95% CI: −7.8 to −2.1) (Table 2, Appendix Table 6). Scenario 2 (stepwise increase to 450) showed a 10% reduction (RR: 0.90, 95% CI: 0.85–0.96; E-value 1.45), and Scenario 5 (universal increase to 600) an 11% reduction (RR: 0.89, 95% CI: 0.82–0.97). Even the most conservative upward shift, raising participants below 150 MET-min/week to 150 (Scenario 1), reduced risk by 7% (RR: 0.93, 95% CI: 0.88-0.99). Conversely, the downward scenario of reducing participants at or above 450 MET-min/week to 150 (Scenario 7) increased risk by 9% (RR: 1.09, 95% CI: 1.03–1.16). The stochastic uptake scenarios (Scenarios 9 and 10) did not reduce the risk of poor self-rated oral health (Scenario 9: RR: 0.99, 95% CI: 0.96–1.02; Scenario 10: RR: 0.98, 95% CI: 0.94–1.03).

**Table 2:** Estimated risk ratios for oral health outcomes under hypothetical physical activity scenarios (MET-min/week).

Physical activity scenario	Super Learner			GLM		
	RR	95% CI	E-value	RR	95% CI	E-value
**Poor self-rated oral health**						
1. Raise <150 to 150	0.93	0.88, 0.99	1.34	0.94	0.89, 1.00	1.31
2. Stepwise to 450	0.90	0.85, 0.96	1.45	0.91	0.85, 0.97	1.44
3. Stepwise ladder to 600	0.92	0.87, 0.99	1.38	0.92	0.87, 0.99	1.38
4. Universal floor at 450	0.88	0.82, 0.95	1.53	0.88	0.83, 0.94	1.52
5. Universal floor at 600	0.89	0.82, 0.97	1.49	0.90	0.84, 0.96	1.47
6. Reduce ≥600 to 450	1.05	0.99, 1.12	1.28	1.03	0.97, 1.10	1.22
7. Reduce ≥450 to 150	1.09	1.03, 1.16	1.41	1.08	1.01, 1.15	1.37
8. All to 0 (extreme)	1.03	0.92, 1.16	1.21	1.04	0.97, 1.12	1.25
9. 25% uptake of <150 to 600	0.99	0.96, 1.02	1.11	0.99	0.97, 1.02	1.08
10. 50% uptake of <150 to 600	0.98	0.94, 1.03	1.14	1.00	0.95, 1.04	1.07
**Any masticatory function impairment (primary)**						
1. Raise <150 to 150	0.94	0.89, 1.00	1.32	0.94	0.89, 1.00	1.31
2. Stepwise to 450	1.01	0.95, 1.07	1.11	1.01	0.95, 1.07	1.10
3. Stepwise ladder to 600	1.03	0.97, 1.10	1.22	1.03	0.97, 1.09	1.19
4. Universal floor at 450	0.97	0.90, 1.04	1.22	0.98	0.93, 1.05	1.14
5. Universal floor at 600	0.98	0.91, 1.05	1.18	0.99	0.93, 1.05	1.11
6. Reduce ≥600 to 450	1.11	1.05, 1.18	1.46	1.14	1.08, 1.21	1.55
7. Reduce ≥450 to 150	1.18	1.12, 1.25	1.64	1.19	1.12, 1.27	1.66
8. All to 0 (extreme)	1.21	1.10, 1.33	1.71	1.20	1.12, 1.28	1.69
9. 25% uptake of <150 to 600	0.97	0.94, 0.99	1.22	0.97	0.95, 1.00	1.19
10. 50% uptake of <150 to 600	0.94	0.90, 0.99	1.31	0.95	0.91, 0.99	1.28
**Any masticatory function impairment, strict (sensitivity)**						
1. Raise <150 to 150	0.91	0.84, 0.98	1.43	0.91	0.84, 0.98	1.43
2. Stepwise to 450	1.00	0.92, 1.08	1.06	0.99	0.92, 1.07	1.10
3. Stepwise ladder to 600	0.99	0.91, 1.07	1.13	0.99	0.91, 1.07	1.12
4. Universal floor at 450	0.94	0.86, 1.03	1.32	0.95	0.88, 1.03	1.27
5. Universal floor at 600	0.90	0.82, 1.00	1.45	0.93	0.85, 1.01	1.37
6. Reduce ≥600 to 450	1.20	1.12, 1.28	1.69	1.22	1.14, 1.30	1.73
7. Reduce ≥450 to 150	1.21	1.13, 1.30	1.72	1.25	1.15, 1.35	1.80
8. All to 0 (extreme)	1.26	1.14, 1.39	1.84	1.26	1.16, 1.37	1.84
9. 25% uptake of <150 to 600	0.96	0.92, 0.99	1.27	0.96	0.93, 1.00	1.24
10. 50% uptake of <150 to 600	0.93	0.88, 0.99	1.34	0.94	0.89, 1.00	1.32

Abbreviations: RR, risk ratio; CI, confidence interval.

Footnote: Estimates are pooled across 10 imputed datasets Super Learner used generalized linear models (GLM), generalized additive models (GAM), and penalized regression via elastic net (GLMNET) as candidate learners. Scenarios 1–5 and 9–10 are upward shifts (capped at stratum-specific P90); Scenarios 6–8 are downward shifts. Adjusted for sex, ethnicity, education, housing type, age, smoking status, diabetes mellitus, cardiovascular disease, cerebrovascular disease, and social support.

For the primary masticatory function impairment outcome, the pattern differed. Deterministic upward scenarios (Scenarios 2–5) did not reduce the risk of masticatory function impairment, while Scenario 1 (raising those below 150 MET-min/week to 150) showed an imprecise reduction (RR: 0.94, 95% CI: 0.89–1.00). In contrast, the stochastic uptake scenarios, where 25% or 50% of participants below 150 MET-min/week were raised toward 600, produced reductions in masticatory function impairment (Scenario 9: RR: 0.97, 95% CI: 0.94–0.99; Scenario 10: RR: 0.94, 95% CI: 0.90–0.99; E-value 1.31). The downward scenarios showed a clear dose-response pattern: reducing those at or above 600 to 450 MET-min/week increased the risk of masticatory function impairment by 11% (Scenario 6: RR: 1.11, 95% CI: 1.05–1.18), reducing those at or above 450 to 150 increased risk by 18% (Scenario 7: RR: 1.18, 95% CI: 1.12–1.25), and eliminating all physical activity increased risk by 21% (Scenario 8: RR: 1.21, 95% CI: 1.10-1.33; E-value 1.71) (Table 2, Appendix Table 5).

The stricter masticatory function impairment definition produced generally consistent but stronger effects (Table 2). Scenario 1 showed a 9% reduction in masticatory function impairment (RR: 0.91, 95% CI: 0.84–0.98; E-value 1.43). The downward scenarios showed larger risk increases, with the extreme counterfactual (Scenario 8) reaching a risk ratio of 1.26 (95% CI: 1.14–1.39; E-value 1.84), suggesting that the protective effect of physical activity may be more pronounced at higher levels of masticatory impairment. Attrition sensitivity analyses under best- and worst-case assumptions for participants censored between Waves 2 and 3 (*n* = 1164) showed that the direction of effects persisted (Appendix Table 6).

E-values for the observed effects ranged modestly from 1.31 to 1.84, indicating that an unmeasured confounder with both physical activity and oral health outcomes by a risk ratio of at least this magnitude would be required to explain away these findings. Super Learner and GLM estimators showed close concordance across all scenarios and outcomes, supporting the robustness of the doubly-robust estimation approach. Positivity diagnostics were acceptable across all 10 scenarios and three outcomes. No cumulative density ratios approached extreme values (i.e., below 0.01 or exceeded 100), and effective sample size proportions ranged from 0.84 to 0.96. Scenarios involving larger counterfactual deviations, such as Scenarios 5 and 8, produced wider density ratio distributions but did not indicate practical positivity violations (Appendix Table 7, Appendix Fig. 2).

## 4 Discussion

This study estimated that increasing physical activity at the population level produced modest reduction in the risk of poor self-rated oral health, while reducing physical activity increased masticatory function impairment in a dose-response manner. The largest reduction in the risk of poor self-rated oral health was observed when all participants accumulated at least 450 MET-min/week, corresponding to approximately 5 fewer cases of poor self-rated oral health per 100 older adults. No additional benefit was observed beyond this, suggesting a plateau in the protective effect for self-rated oral health. For masticatory function impairment, the effects were asymmetric: deterministic upward shifts did not consistently reduce risk, whereas downward shifts produced progressively larger increases, with the elimination of all activity raising risk by 21%. The significance of the stochastic uptake scenarios for masticatory function impairment suggests that targeting even a fraction of the most inactive older adults for meaningful increases in physical activity could yield population-level benefits.

Effects were robust to the choice of estimator, outcome definition, and extreme attrition assumptions. These findings are consistent with cross-sectional evidence linking higher physical activity to better oral health outcomes. Physical activity has been associated with lower prevalence of dental caries, gingival bleeding, and tooth loss [32], reduced periodontal disease risk [33], and lower edentulism risk [34].

The most striking finding was the asymmetry between the two outcomes: self-rated oral health responded to deterministic upward shifts across multiple scenarios, whereas masticatory function impairment was primarily responsive to downward and stochastic scenarios. This pattern reflects the distinct constructs these measures capture. Self-rated oral health is a subjective, multidimensional indicator encompassing not only clinical oral status but also functional and psychosocial dimensions [35,36], making it more sensitive to general health improvements associated with increased physical activity. In contrast, masticatory function impairment is more directly determined by the number and condition of remaining teeth, prosthetic adequacy, and neuromuscular coordination [37]. Physical activity helps preserve skeletal muscle mass, and sarcopenia is recognized as a systemic process affecting masticatory muscles and reducing bite force [38]. Evidence from two prospective cohorts further suggests a bidirectional relationship between oral and physical function, with poor oral health markers—including self-rated oral health—predicting subsequent declines in muscle strength and physical performance [39]. The stronger and more consistent effects of downward scenarios on masticatory function impairment align with this mechanism: declines in physical function may accelerate deterioration in masticatory muscle performance. However, masticatory function depends on tooth condition, prosthetic rehabilitation, and muscle function. Physical activity can influence only the latter, and cannot compensate for structural tooth loss or inadequate rehabilitation. Supporting this interpretation, a Mendelian randomization study found no causal effect of genetically predicted physical activity on periodontitis risk [40], indicating that the relationship between physical activity and oral health likely operates primarily through neuromuscular, behavioural, and functional pathways rather than periodontal inflammation.

Studies of physical activity and oral health have traditionally relied on conventional regression using the exposure measured at a single time point. Such analyses estimate differences in oral health between individuals at different observed levels of physical activity, rather than the effects of specified changes in activity. In contrast, LMTPs allow the effects of specified interventions on a continuous, time-varying exposure to be estimated while accommodating repeated measurements of the exposure and relevant confounders over follow-up. This provides a more intervention-based interpretation, addressing what oral health outcomes might be expected under specified changes in population physical activity rather than simply comparing individuals who were observed to have different activity levels.

This study is, to our knowledge, the first to apply a causal inference framework to estimate the effect of physical activity on oral health outcomes. Physical activity is a continuous, time-varying exposure for which static interventions are neither realistic nor well supported by the data. The longitudinal modified treatment policy (LMTP) approach defines interventions that shift each individual’s observed exposure by a feasible amount, thereby respecting the positivity assumption while retaining a causal interpretation [26,41]. The 10 scenarios are not intended as directly implementable interventions but as counterfactuals designed to characterise the dose-response relationship, providing an upper and lower bounds that may inform the scale of benefit or harm policymakers could anticipate from population-level changes in physical activity among older adults.

This study has several limitations. Physical activity was assessed using the Global Physical Activity Questionnaire, which relies on self-report and tends to overestimate higher activity levels, potentially attenuating observed effects [42,43]. Both oral health outcomes were also self-reported, raising the possibility that the observed association between physical activity and oral health is partly confounded by general health awareness or individual reporting tendencies. In addition, oral health outcomes were not measured at Wave 1, which precluded adjustment for baseline oral health status; individuals with pre-existing poor oral health may have been less physically active, introducing potential bias from unmeasured baseline confounding. Attrition over the study period was substantial. Although the LMTP approach accounts for censoring, and sensitivity analyses under extreme attrition assumptions supported the direction of the primary findings, the estimates may not fully reflect the experiences of participants who were lost to follow-up.

The effects of physical activity on oral health may also vary across population subgroups and warrant further investigation. Frailty and sarcopenia may be relevant because muscle strength and function contribute to masticatory performance [39,44,45,53], raising the possibility that the effect of increased physical activity on oral function differs according to underlying physical reserve. Nutritional status may also modify this relationship, given the interrelationships between dietary intake, muscle health, physical activity, and masticatory function [46]. Socially disadvantaged older adults experience a greater burden of oral disease while also facing greater barriers to physical activity and dental care [47,48,54]. Consequently, population-level increases in physical activity may not produce equal benefits across these population subgroups. Examining such heterogeneity would help determine whether the magnitude of benefit differs across these groups, and methods for data-adaptive identification of effect modifiers under varying interventions may facilitate such analyses [49]. Further research should also examine whether physical activity interventions improve clinical oral health indicators using objective measures, and replicate these findings in other ageing populations. Translating any such benefit into practice would additionally require implementation research to determine how activity promotion might be embedded within existing care pathways for older adults [50].

## 5 Conclusions

Increasing physical activity was causally associated with lower risk of poor self-rated oral health in older adults, while reductions in activity were consistently linked to greater masticatory function impairment in a dose-response manner. These findings suggest that promoting physical activity, particularly among the most inactive, may contribute to improved oral health in ageing populations.

## CRediT authorship contribution statement

John Rong Hao Tay: Conceptualisation, Methodology, Formal analysis, Writing - original draft, Writing - review & editing. Gustavo G. Nascimento: Conceptualisation, Methodology, Writing - review & editing. Rahul Malhotra: Resources, Funding acquisition, Methodology, Writing - review & editing. Pedro C. Hallal: Methodology, Writing - review & editing. Marco A. Peres: Supervision, Methodology, Writing - review & editing.

## Ethics approval

THE SIGNS Waves 1 and 2 was approved by the IRB at the National University of Singapore (NUS-IRB Reference Code: B-15-152), and Wave 3 was approved by the IRB at the National University of Singapore (NUS-IRB-2022-627).

## Consent for publication

Not applicable. This article does not contain any identifiable individual person’s data.

## Declaration of the use of generative AI and AI-assisted technologies in scientific writing and in figures, images and artwork

This manuscript used NLP solely for proofreading text that was entirely human-generated. BioRender was used to assist in generating the figure. No other use of AI was made.

## Funding

Waves 1 and 2 of Transitions in Health, Employment, Social Engagement and Inter-Generational Transfers in Singapore (THE SIGNS) Study were supported by Singapore’s Ministry of Health(MOH) under the agreement number MOH-NUS RL2015-053. Wave 3 of THE SIGNS was funded by Singapore's Ministry of Health (MOH) under agreement number NUS-2022-1133and variation agreement number NUS-2024-0586.

## Availability of data and materials

The data that support the findings of this study are available from the Centre for Ageing Research & Education, Duke-NUS Medical School. Restrictions apply to the availability of these data, which were used under license for this study. Data are available from the authors upon reasonable request, with the permission of Duke-NUS Medical School and other relevant stakeholders.

## Declaration of competing interest

The authors declare the following financial interests/personal relationships which may be considered as potential competing interests:

John Rong Hao Tay reports financial support was provided by Government of Singapore Ministry of Health. Rahul Malhotra reports financial support was provided by Government of Singapore Ministry of Health. Marco A. Peres reports financial support was provided by Government of Singapore Ministry of Health. If there are other authors, they declare that they have no known competing financial interests or personal relationships that could have appeared to influence the work reported in this paper.

## Glossary

ADL: Activity of daily living
CI: Confidence interval
GLM: Generalised linear model
GPAQ: Global Physical Activity Questionnaire
IRB: Institutional Review Board
LMTP: Longitudinal modified treatment policy
MET: Metabolic equivalent of task
N: Number
RD: Risk difference
RR: Risk ratio
SD: Standard deviation
THE SIGNS/TSS: Transitions in Health, Employment, Social Engagement and Inter-generational Transfers in Singapore
TMLE: Targeted maximum likelihood estimation
WHO: World Health Organization
