# Supplementary material for: The effect of increases in physical activity on oral health and function in older adults in Singapore: A longitudinal causal inference study
Source: J Nutr Health Aging. 2026 Aug 28;30(11):100968. doi: 10.1016/j.jnha.2026.100968 (PMC13545370; doi:10.1016/j.jnha.2026.100968)
Supplement: Supplementary file 1 [file mmc1.docx]

**Appendix S1**

Physical activity reports followed the WHO Global Physical Activity Questionnaire (GPAQ). Participants reported days/week and minutes/day of activity across three domains:

1. Work-related activity (vigorous or moderate)
2. Active transport (walking or cycling)
3. Leisure-time activity (vigorous or moderate)

Activities were assigned fixed WHO metabolic equivalent (MET) values:

- Vigorous = 8.0 METs
- Moderate = 4.0 METs
- Transport walking/cycling = 4.0 METs

Weekly MET-minutes were computed as: “MET-min/week” = (8× “vigorous minutes/week”) + (4× “moderate/transport minutes/week”. Light-intensity activities were not included.

Quality checks were applied at both waves 1 and 2 [1]. Physical activity was treated as invalid (i.e., missing data) if any of the following occurred:

1. Any sub-domain >16 hours/day
2. Days/week >7
3. Internal inconsistencies, including:
   - days = 0 but minutes > 0
   - days > 0 but time missing
   - minute fields ≥60
4. Total activity >24 hours/day across all sub-domains

The 95th percentile of total MET-minutes/week was also used as a common upper bound. Participants were excluded if:

- MET-min/week at wave 1 > P95, or
- MET-min/week at wave 2 > P95.

This approach avoids wave-specific cut-offs susceptible to differential reporting error and treats extremely high values as implausible.

**Appendix S2**

Socio-demographics assessed at baseline (or wave 1)—sex (male/female), ethnicity (Chinese/non-Chinese), education (primary or below/above primary), housing type (1-2 room government-built housing/3-room government-built housing/4-5 room government-built housing or private home), and smoking (current/never or former)—were treated as time-invariant confounders. Baseline health and psychosocial characteristics—including diabetes mellitus, cardiovascular disease, cancer, cerebrovascular disease, clinically significant depressive symptoms, and social support—were also treated as time-invariant because they precede the exposure and may influence both physical activity and oral health outcomes. Medical conditions were self-reported (yes/no). Social support was measured using a modified Lubben Social Network Scale-Revised (score range 0-60), with higher scores indicating greater perceived social support [2, 3]. Depressive symptoms were assessed using the 11-item Center for Epidemiologic Studies Depression Scale (CES-D) [4]. Items were rated on a three-point frequency scale, yielding a total score from 0 to 22; higher scores indicate more depressive symptoms. Following established cut-offs, scores ≥7 were classified as clinically significant depressive symptoms [5, 6].

Wave 2 values of these variables (including age, smoking status, diabetes mellitus, cardiovascular disease, cerebrovascular disease, cancer, depressive symptoms, and social support) were not included as time-varying confounders because they may lie on the causal pathway between physical activity and oral health outcomes. Conditioning on post-exposure values of these variables would block mediated effects and underestimate the total causal effect of physical activity (Figure 1).

**Appendix Table 1.** Assessment of oral health outcomes.

**Panel A.** Food groups used for the assessment of perceived masticatory function.

| Food group | Representative foods | Toughness |
| --- | --- | --- |
| Group 1 | Ikan bilis (dried anchovies) or shredded dried squid | Hardest |
| Group 2 | Mutton curry, dried mango, or fresh carrots | ↓ |
| Group 3 | Bak-kwa (barbecued sweet pork jerky), bread with crust (not toasted), steamed kang kong (water spinach), chicken satay (skewered grilled chicken), or raw cucumber | ↓ |
| Group 4 | Thai rice, fried fish balls (minced fish formed into chewy spheres), or wanton (dumpling) noodles | ↓ |
| Group 5 | Bananas, ripe papaya, or hard-boiled egg | ↓ |
| Group 6 | Unable to chew the foods listed in Group 5 | Softest / unable |

Participants were asked: “The following foods are ordered from hardest to softest to chew. What is the hardest group you are able to bite and chew?”

Food groups were categorized based on measured toughness and masticatory muscle activity (Nasu and Saito 2006). Toughness values were obtained using a texturometer applied to commonly consumed local foods to aid participant comprehension, and perceived masticatory ability was coded on a six-level scale (Groups 1–6, increasing difficulty).

Primary outcome: Group 1 = no difficulty; Groups 2–6 = any difficulty.

Sensitivity analysis: Groups 1–2 = no difficulty; Groups 3–6 = any difficulty.

**Panel B.** Self-reported oral health

Self-rated oral health was assessed using the question:

“Over the past 6 months, how would you rate your state of oral health?” with response categories:

1 = Excellent, 2 = Very good, 3 = Good, 4 = Fair, 5 = Poor

For analysis, responses were dichotomized as good oral health (Excellent/Very good/Good); and poor oral health (Fair/Poor).

**Appendix Table 2.** Baseline (Wave 1) characteristics prior to multiple imputation.

| Characteristic | Overall (N = 3981) |
| --- | --- |
| Age, years | 73.2 (8.5) |
| **Sex** |  |
| Male | 1744 (43.8) |
| Female | 2237 (56.2) |
| **Ethnicity** |  |
| Chinese | 2935 (73.7) |
| Non-Chinese | 1046 (26.3) |
| **Education** |  |
| Primary school or below | 2472 (62.1) |
| Above primary school | 1500 (37.7) |
| *Missing* | *9 (0.2)* |
| **Housing type** |  |
| 1–2 room government-built | 338 (8.5) |
| 3 room government-built | 968 (24.3) |
| 4–5 room government-built/private | 2673 (67.1) |
| *Missing* | *2 (0.1)* |
| **Smoking status** |  |
| Never or former | 3550 (89.2) |
| Current | 412 (10.3) |
| *Missing* | *19 (0.5)* |
| **ADL limitation** |  |
| None | 3463 (87.0) |
| ≥1 limitation | 514 (12.9) |
| *Missing* | *4 (0.1)* |
| **Social network scale (Lubben, 0–60)** | 26.1 (11.1) |
| *Missing* | *501 (12.6)* |
| **Clinically significant depressive symptoms** |  |
| No | 1631 (41.0) |
| Yes | 238 (6.0) |
| *Missing* | *2112 (53.1)* |
| **Diabetes mellitus** |  |
| No | 2872 (72.1) |
| Yes | 1102 (27.7) |
| *Missing* | *7 (0.2)* |
| **Cardiovascular disease** |  |
| No | 3370 (84.7) |
| Yes | 601 (15.1) |
| *Missing* | *10 (0.3)* |
| **Cerebrovascular disease** |  |
| No | 3752 (94.2) |
| Yes | 224 (5.6) |
| *Missing* | *5 (0.1)* |
| **Cancer** |  |
| No | 3784 (95.1) |
| Yes | 192 (4.8) |
| *Missing* | *5 (0.1)* |
| **Physical activity, MET-min/week** |  |
| Mean (SD) | 517.9 (1004.6) |
| <150 | 2585 (64.9) |
| 150–599 | 363 (9.1) |
| ≥600 (meeting WHO guideline) | 1033 (25.9) |

Abbreviations: ADL, activities of daily living; CES-D, Center for Epidemiologic Studies Depression Scale; MET, metabolic equivalent of task; N, number; SD, standard deviation; WHO, World Health Organization.

Footnote: Values are N (%) for categorical variables and Mean (SD) for continuous variables. Statistics are pooled across 10 imputed datasets. Clinically significant depressive symptoms defined as CES-D 11-item score ≥7 (Yokoyama et al. 2010; Malhotra et al. 2011).

**Appendix Table 3.** Baseline (Wave 1) characteristics by baseline physical activity category, multiple imputed data.

| Characteristic | <150 MET-min/week (n = 2585) | 150–599 MET-min/week (n = 363) | ≥600 MET-min/week (n = 1033) |
| --- | --- | --- | --- |
| Age, years | 74.0 (8.8) | 73.3 (8.2) | 71.3 (7.4) |
| **Sex** |  |  |  |
| Male | 1102 (42.6) | 158 (43.5) | 484 (46.9) |
| Female | 1483 (57.4) | 205 (56.5) | 549 (53.1) |
| **Ethnicity** |  |  |  |
| Chinese | 1869 (72.3) | 259 (71.3) | 807 (78.1) |
| Non-Chinese | 716 (27.7) | 104 (28.7) | 226 (21.9) |
| **Education** |  |  |  |
| Primary school or below | 1663 (64.3) | 237 (65.3) | 581 (56.2) |
| Above primary school | 922 (35.7) | 126 (34.7) | 452 (43.8) |
| **Housing type** |  |  |  |
| 1–2 room government-built | 206 (8.0) | 42 (11.6) | 90 (8.7) |
| 3 room government-built | 630 (24.4) | 81 (22.3) | 257 (24.9) |
| 4–5 room government-built/private | 1748 (67.6) | 240 (66.1) | 686 (66.4) |
| **Smoking status** |  |  |  |
| Never or former | 2333 (90.2) | 320 (88.2) | 915 (88.6) |
| Current | 252 (9.8) | 43 (11.8) | 118 (11.4) |
| **ADL limitation** |  |  |  |
| None | 2124 (82.2) | 339 (93.4) | 1000 (96.8) |
| ≥1 limitation | 461 (17.8) | 24 (6.6) | 33 (3.2) |
| **Social network scale (Lubben, 0–60)** | 25.0 (11.2) | 25.9 (11.2) | 27.2 (10.7) |
| **Clinically significant depressive symptoms** |  |  |  |
| No | 2194 (84.9) | 327 (90.0) | 955 (92.5) |
| Yes | 391 (15.1) | 36 (10.0) | 78 (7.5) |
| **Diabetes mellitus** |  |  |  |
| No | 1816 (70.2) | 258 (71.1) | 802 (77.6) |
| Yes | 769 (29.8) | 105 (28.9) | 231 (22.4) |
| **Cardiovascular disease** |  |  |  |
| No | 2161 (83.6) | 311 (85.7) | 907 (87.8) |
| Yes | 424 (16.4) | 52 (14.3) | 126 (12.2) |
| **Cerebrovascular disease** |  |  |  |
| No | 2419 (93.6) | 341 (93.9) | 997 (96.5) |
| Yes | 166 (6.4) | 22 (6.1) | 36 (3.5) |
| **Cancer** |  |  |  |
| No | 2464 (95.3) | 341 (93.9) | 984 (95.3) |
| Yes | 121 (4.7) | 22 (6.1) | 49 (4.7) |
| **Physical activity, MET-min/week** |  |  |  |
| Mean (SD) | 1.6 (13.1) | 382.1 (126.1) | 1857.8 (1189.3) |

Abbreviations: ADL, activities of daily living; CES-D, Center for Epidemiologic Studies Depression Scale; MET, metabolic equivalent of task; N, number; SD, standard deviation; WHO, World Health Organization.

Footnote: Values are N (%) for categorical variables and Mean (SD) for continuous variables. Statistics are pooled across 10 imputed datasets. Clinically significant depressive symptoms defined as CES-D 11-item score ≥7 (Yokoyama et al. 2010; Malhotra et al. 2011).

**Appendix Table 4.** Missing data by variable and wave, prior to multiple imputation.

| Variable | Wave 1 (N = 3981) | Wave 2 (N = 2383) | Wave 3 (N = 1219) |
| --- | --- | --- | --- |
| Age, years | 0 (0.0) | — | — |
| Sex | 0 (0.0) | — | — |
| Ethnicity | 0 (0.0) | — | — |
| Education | 9 (0.2) | — | — |
| Housing type | 2 (0.1) | — | — |
| Smoking status | 19 (0.5) | — | — |
| ADL limitation | 4 (0.1) | 0 (0.0) | — |
| Social support (Lubben) | 501 (12.6) | — | — |
| Depressive symptoms (CES-D ≥7) | 2112 (53.1) | — | — |
| Diabetes mellitus | 7 (0.2) | — | — |
| Cardiovascular disease | 10 (0.3) | — | — |
| Cerebrovascular disease | 5 (0.1) | — | — |
| Cancer | 5 (0.1) | — | — |
| Physical activity (MET-min/week) | 0 (0.0) | 0 (0.0) | — |
| Self-rated oral health | — | — | 3 (0.2) |
| Masticatory function impairment | — | — | 6 (0.5) |
| Censored (lost to follow-up) | — | 1598 (40.1) | 1164 (48.8) |

Abbreviations: ADL, activities of daily living; CES-D, Center for Epidemiologic Studies Depression Scale; MET, metabolic equivalent of task; N, number.

Footnote: Values are N (%) missing. (—) indicates the variable was not measured or not applicable at that wave. Wave 2 and 3 denominators reflect participants observed at the respective wave. The CES-D was administered to a random half of the sample at Wave 1 to reduce respondent burden, accounting for the majority of its missingness.

**Appendix Table 5.** Estimated risk differences for oral health outcomes under hypothetical physical activity scenarios.

|  | Super Learner | | | GLM | | |
| --- | --- | --- | --- | --- | --- | --- |
| **Scenario** | **RD** | **95% CI** | **E-value** | **RD** | **95% CI** | **E-value** |
| **Poor self-rated oral health** |  |  |  |  |  |  |
| 1. Raise <150 to 150 | -0.027 | -0.050, -0.003 | 1.34 | -0.023 | -0.047, 0.001 | 1.31 |
| 2. Stepwise to 450 | -0.040 | -0.065, -0.014 | 1.45 | -0.038 | -0.064, -0.013 | 1.44 |
| 3. Stepwise ladder to 600 | -0.031 | -0.057, -0.005 | 1.38 | -0.031 | -0.057, -0.005 | 1.38 |
| 4. Universal floor at 450 | -0.049 | -0.078, -0.021 | 1.53 | -0.048 | -0.074, -0.022 | 1.52 |
| 5. Universal floor at 600 | -0.044 | -0.075, -0.013 | 1.49 | -0.042 | -0.069, -0.015 | 1.47 |
| 6. Reduce ≥600 to 450 | 0.021 | -0.005, 0.046 | 1.28 | 0.014 | -0.012, 0.039 | 1.22 |
| 7. Reduce ≥450 to 150 | 0.037 | 0.013, 0.061 | 1.41 | 0.032 | 0.004, 0.061 | 1.37 |
| 8. All to 0 (extreme) | 0.013 | -0.037, 0.062 | 1.21 | 0.017 | -0.014, 0.047 | 1.25 |
| 9. 25% uptake of <150 to 600 | -0.004 | -0.016, 0.007 | 1.11 | -0.002 | -0.014, 0.010 | 1.08 |
| 10. 50% uptake of <150 to 600 | -0.006 | -0.024, 0.012 | 1.14 | -0.002 | -0.019, 0.016 | 1.07 |
| **Any masticatory function impairment (primary)** |  |  |  |  |  |  |
| 1. Raise <150 to 150 | -0.025 | -0.049, -0.001 | 1.32 | -0.024 | -0.048, -0.001 | 1.31 |
| 2. Stepwise to 450 | 0.004 | -0.021, 0.029 | 1.11 | 0.004 | -0.022, 0.030 | 1.10 |
| 3. Stepwise ladder to 600 | 0.014 | -0.011, 0.040 | 1.22 | 0.011 | -0.015, 0.037 | 1.19 |
| 4. Universal floor at 450 | -0.014 | -0.042, 0.015 | 1.22 | -0.007 | -0.033, 0.019 | 1.14 |
| 5. Universal floor at 600 | -0.010 | -0.042, 0.021 | 1.18 | -0.004 | -0.031, 0.022 | 1.11 |
| 6. Reduce ≥600 to 450 | 0.047 | 0.021, 0.074 | 1.46 | 0.061 | 0.034, 0.087 | 1.55 |
| 7. Reduce ≥450 to 150 | 0.078 | 0.053, 0.102 | 1.64 | 0.081 | 0.052, 0.110 | 1.66 |
| 8. All to 0 (extreme) | 0.090 | 0.044, 0.136 | 1.71 | 0.086 | 0.055, 0.117 | 1.69 |
| 9. 25% uptake of <150 to 600 | -0.013 | -0.025, -0.002 | 1.22 | -0.011 | -0.023, 0.000 | 1.19 |
| 10. 50% uptake of <150 to 600 | -0.024 | -0.042, -0.006 | 1.31 | -0.020 | -0.037, -0.003 | 1.28 |
| **Any masticatory function impairment, strict (sensitivity)** |  |  |  |  |  |  |
| 1. Raise <150 to 150 | -0.025 | -0.046, -0.005 | 1.43 | -0.025 | -0.046, -0.004 | 1.43 |
| 2. Stepwise to 450 | -0.001 | -0.022, 0.020 | 1.06 | -0.003 | -0.024, 0.019 | 1.10 |
| 3. Stepwise ladder to 600 | -0.004 | -0.026, 0.018 | 1.13 | -0.003 | -0.025, 0.019 | 1.12 |
| 4. Universal floor at 450 | -0.016 | -0.040, 0.008 | 1.32 | -0.013 | -0.035, 0.010 | 1.27 |
| 5. Universal floor at 600 | -0.027 | -0.053, 0.000 | 1.45 | -0.020 | -0.043, 0.002 | 1.37 |
| 6. Reduce ≥600 to 450 | 0.056 | 0.036, 0.077 | 1.69 | 0.061 | 0.039, 0.082 | 1.73 |
| 7. Reduce ≥450 to 150 | 0.059 | 0.039, 0.080 | 1.72 | 0.069 | 0.045, 0.093 | 1.80 |
| 8. All to 0 (extreme) | 0.073 | 0.042, 0.105 | 1.84 | 0.074 | 0.048, 0.100 | 1.84 |
| 9. 25% uptake of <150 to 600 | -0.012 | -0.023, -0.002 | 1.27 | -0.011 | -0.021, 0.000 | 1.24 |
| 10. 50% uptake of <150 to 600 | -0.018 | -0.034, -0.002 | 1.34 | -0.016 | -0.031, -0.001 | 1.32 |

Abbreviations: RR, risk ratio; CI, confidence interval.

Footnote: Estimates are pooled across 10 imputed datasets Super Learner used generalized linear models (GLM), generalized additive models (GAM), and penalized regression via elastic net (GLMNET) as candidate learners. Scenarios 1–5 and 9–10 are upward shifts (capped at stratum-specific P90); Scenarios 6–8 are downward shifts.

**Appendix Table 6.** Sensitivity analysis for non-random attrition at Wave 3 under best- and worst-case assumptions.

|  | Poor self-rated oral health | | Any masticatory function impairment (primary) | | Any masticatory function impairment, strict (sensitivity) | |
| --- | --- | --- | --- | --- | --- | --- |
| **Scenario** | **Best case RR (95% CI)** | **Worst case RR (95% CI)** | **Best case RR (95% CI)** | **Worst case RR (95% CI)** | **Best case RR (95% CI)** | **Worst case RR (95% CI)** |
| 1. Raise <150 to 150 | 1.01 (0.93, 1.11) | 0.97 (0.94, 0.99) | 1.03 (0.95, 1.12) | 0.97 (0.95, 0.99) | 0.95 (0.84, 1.08) | 0.96 (0.93, 0.98) |
| 2. Stepwise to 450 | 0.93 (0.84, 1.02) | 0.97 (0.95, 0.99) | 1.05 (0.96, 1.15) | 0.99 (0.97, 1.01) | 1.00 (0.88, 1.13) | 0.98 (0.96, 1.01) |
| 3. Stepwise ladder to 600 | 0.98 (0.89, 1.08) | 0.97 (0.95, 0.99) | 1.09 (1.00, 1.20) | 0.99 (0.97, 1.02) | 1.01 (0.88, 1.15) | 0.97 (0.95, 1.00) |
| 4. Universal floor at 450 | 0.94 (0.85, 1.04) | 0.96 (0.94, 0.98) | 1.05 (0.96, 1.15) | 0.98 (0.96, 1.00) | 0.98 (0.86, 1.12) | 0.96 (0.94, 0.99) |
| 5. Universal floor at 600 | 1.00 (0.90, 1.10) | 0.96 (0.94, 0.98) | 1.10 (1.00, 1.20) | 0.98 (0.96, 1.00) | 0.99 (0.86, 1.13) | 0.95 (0.93, 0.98) |
| 6. Reduce ≥600 to 450 | 0.86 (0.73, 1.01) | 1.06 (1.03, 1.10) | 0.96 (0.83, 1.12) | 1.09 (1.05, 1.12) | 1.10 (0.92, 1.30) | 1.12 (1.08, 1.16) |
| 7. Reduce ≥450 to 150 | 0.82 (0.66, 1.00) | 1.10 (1.06, 1.14) | 0.91 (0.76, 1.10) | 1.12 (1.08, 1.16) | 1.00 (0.80, 1.26) | 1.15 (1.10, 1.20) |
| 8. All to 0 (extreme) | 0.76 (0.62, 0.95) | 1.11 (1.07, 1.15) | 0.88 (0.72, 1.06) | 1.13 (1.09, 1.17) | 0.98 (0.78, 1.24) | 1.17 (1.12, 1.22) |
| 9. 25% uptake of <150 to 600 | 1.02 (0.97, 1.07) | 0.99 (0.98, 1.00) | 0.99 (0.95, 1.04) | 0.99 (0.98, 1.00) | 0.96 (0.91, 1.02) | 0.99 (0.97, 1.00) |
| 10. 50% uptake of <150 to 600 | 1.04 (0.97, 1.11) | 0.99 (0.97, 1.00) | 0.99 (0.93, 1.05) | 0.98 (0.96, 0.99) | 0.95 (0.87, 1.04) | 0.97 (0.96, 0.99) |

Abbreviations: CI, confidence interval; RR, risk ratio.

Footnote: Best case: all participants censored before Wave 3 (n = 1,164) assumed to have not developed outcome of interest at Wave 3. Worst case: all censored participants assumed to have developed the outcome.

**Appendix Table 7.** Positivity diagnostics: cumulative density ratio distributions by scenario and outcome.

|  | Cumulative density ratio | | | Extremes | |  |
| --- | --- | --- | --- | --- | --- | --- |
| **Scenario** | **Min** | **1^st^ percentile** | **99th percentile** | **% < 0.01** | **% > 100** | **ESS proportion** |
| **Poor self-rated oral health** |  |  |  |  |  |  |
| 1. Raise <150 to 150 | 1.28 | 1.44 | 4.98 | 0.0 | 0.0 | 0.91 |
| 2. Stepwise to 450 | 1.29 | 1.47 | 4.87 | 0.0 | 0.0 | 0.92 |
| 3. Stepwise ladder to 600 | 1.31 | 1.52 | 4.73 | 0.0 | 0.0 | 0.93 |
| 4. Universal floor at 450 | 1.17 | 1.48 | 9.38 | 0.0 | 0.0 | 0.86 |
| 5. Universal floor at 600 | 1.39 | 1.67 | 11.87 | 0.0 | 0.0 | 0.86 |
| 6. Reduce ≥600 to 450 | 2.35 | 2.60 | 11.14 | 0.0 | 0.0 | 0.88 |
| 7. Reduce ≥450 to 150 | 1.78 | 2.03 | 6.63 | 0.0 | 0.0 | 0.95 |
| 8. All to 0 (extreme) | 2.31 | 2.50 | 8.52 | 0.0 | 0.0 | 0.89 |
| 9. 25% uptake of <150 to 600 | 1.18 | 1.36 | 5.26 | 0.0 | 0.0 | 0.90 |
| 10. 50% uptake of <150 to 600 | 1.06 | 1.24 | 6.63 | 0.0 | 0.0 | 0.84 |
| **Any masticatory function impairment (primary)** |  |  |  |  |  |  |
| 1. Raise <150 to 150 | 1.29 | 1.45 | 4.95 | 0.0 | 0.0 | 0.92 |
| 2. Stepwise to 450 | 1.29 | 1.47 | 4.86 | 0.0 | 0.0 | 0.92 |
| 3. Stepwise ladder to 600 | 1.33 | 1.51 | 4.74 | 0.0 | 0.0 | 0.93 |
| 4. Universal floor at 450 | 1.17 | 1.47 | 9.40 | 0.0 | 0.0 | 0.86 |
| 5. Universal floor at 600 | 1.39 | 1.70 | 11.99 | 0.0 | 0.0 | 0.85 |
| 6. Reduce ≥600 to 450 | 2.39 | 2.57 | 11.23 | 0.0 | 0.0 | 0.88 |
| 7. Reduce ≥450 to 150 | 1.83 | 2.13 | 6.58 | 0.0 | 0.0 | 0.96 |
| 8. All to 0 (extreme) | 2.40 | 2.54 | 8.52 | 0.0 | 0.0 | 0.91 |
| 9. 25% uptake of <150 to 600 | 1.14 | 1.35 | 5.27 | 0.0 | 0.0 | 0.90 |
| 10. 50% uptake of <150 to 600 | 1.05 | 1.23 | 6.64 | 0.0 | 0.0 | 0.84 |
| **Any masticatory function impairment, strict (sensitivity)** |  |  |  |  |  |  |
| 1. Raise <150 to 150 | 1.26 | 1.44 | 4.96 | 0.0 | 0.0 | 0.92 |
| 2. Stepwise to 450 | 1.27 | 1.48 | 4.87 | 0.0 | 0.0 | 0.92 |
| 3. Stepwise ladder to 600 | 1.28 | 1.52 | 4.74 | 0.0 | 0.0 | 0.93 |
| 4. Universal floor at 450 | 1.14 | 1.44 | 9.34 | 0.0 | 0.0 | 0.86 |
| 5. Universal floor at 600 | 1.39 | 1.68 | 11.97 | 0.0 | 0.0 | 0.85 |
| 6. Reduce ≥600 to 450 | 2.35 | 2.56 | 11.18 | 0.0 | 0.0 | 0.88 |
| 7. Reduce ≥450 to 150 | 1.83 | 2.13 | 6.58 | 0.0 | 0.0 | 0.96 |
| 8. All to 0 (extreme) | 2.51 | 2.71 | 8.54 | 0.0 | 0.0 | 0.91 |
| 9. 25% uptake of <150 to 600 | 1.16 | 1.36 | 5.27 | 0.0 | 0.0 | 0.90 |
| 10. 50% uptake of <150 to 600 | 1.06 | 1.24 | 6.62 | 0.0 | 0.0 | 0.84 |

Abbreviations: ESS, effective sample size; MET, metabolic equivalent of task.

Footnote: Cumulative density ratios quantify how different each participant's shifted exposure is from their observed exposure, with values closer to 1 indicating a smaller shift. Large values suggest that the intervention assigns an exposure level that is unlikely given the participant's observed profile. ESS proportion is the effective sample size as a proportion of the total uncensored sample, with values closer to 1 indicating better support for the assumed intervention. Diagnostics are averaged across 10 imputed datasets.

**Appendix Table 8.** Stratum-specific 90th percentile caps for physical activity interventions.

| Age band | ADL limitation | Wave 1 P90 (MET-min/wk) | Wave 2 P90 (MET-min/wk) |
| --- | --- | --- | --- |
| 60–79 | None | 2000 | 4200 |
| 60–79 | ≥1 limitation | 572 | 1350 |
| ≥80 | None | 1560 | 3360 |
| ≥80 | ≥1 limitation | 0 | 536 |

Abbreviations: ADL, activities of daily living; MET, metabolic equivalent of task.

Footnote: P90: 90th percentile of observed MET-minutes/week within each age band × ADL stratum. Used as the upper cap for intervention-assigned activity levels in all upward shift scenarios.

**Appendix Figure 1**. Flowchart of participant selection and follow-up from Wave 1 (2016-17) to Wave 3 (2023-24).

**Appendix Figure 2.** Distribution of cumulative density ratios by scenario, for the any masticatory function impairment (primary) outcome. Density ratio distributions were comparable across all three outcomes (Appendix Table S7).

**Appendix References**

1.	World Health Organization. GPAQ Analysis Guide 2021 [Available from: <https://www.who.int/teams/noncommunicable-diseases/surveillance/systems-tools/physical-activity-surveillance>.

2.	Chan A, Malhotra R, Manap N, Ting Y, Visaria A, Cheng G-L, et al. Transitions in Health, Employment, Social Engagement And Intergenerational Transfers In Singapore Study (THE SIGNS Study) – I: Descriptive Statistics and Analysis of Key Aspects of Successful Ageing. Singapore: Centre for Ageing Research and Education, Duke-NUS Medical School.; 2018.

3.	Lubben J, Blozik E, Gillmann G, Iliffe S, von Renteln Kruse W, Beck JC, et al. Performance of an abbreviated version of the Lubben Social Network Scale among three European community-dwelling older adult populations. Gerontologist. 2006;46(4):503-13.

4.	Kohout FJ, Berkman LF, Evans DA, Cornoni-Huntley J. Two shorter forms of the CES-D (Center for Epidemiological Studies Depression) depression symptoms index. J Aging Health. 1993;5(2):179-93.

5.	Malhotra C, Chan A, Malhotra R, Ostbye T. Fifteen Dimensions of Health among Community-Dwelling Older Singaporeans. Curr Gerontol Geriatr Res. 2011;2011:128581.

6.	Yokoyama E, Kaneita Y, Saito Y, Uchiyama M, Matsuzaki Y, Tamaki T, et al. Association between depression and insomnia subtypes: a longitudinal study on the elderly in Japan. Sleep. 2010;33(12):1693-702.
